# Exogenous Promoter Triggers APETALA3 Silencing through RNA-Directed DNA Methylation Pathway in *Arabidopsis*

**DOI:** 10.3390/ijms20184478

**Published:** 2019-09-11

**Authors:** Benqi Wang, Jie Liu, Lei Chu, Xue Jing, Huadong Wang, Jian Guo, Bin Yi

**Affiliations:** National Key Laboratory of Crop Genetic Improvement, National Center of Rapeseed Improvement, Huazhong Agricultural University, Wuhan 430070, China

**Keywords:** *AP3* promoter, methylation, RdDM, transcriptional silencing

## Abstract

The development of floral organs plays a vital role in plant reproduction. In our research, the *APETALA3* (*AP3*) promoter-transgenic lines showed abnormal developmental phenotypes in stamens and petals. The aim of this study is to understand the molecular mechanisms of the morphological defects in transgenic plants. By performing transgenic analysis, it was found that the *AP3*-promoted genes and the vector had no relation to the morphological defects. Then, we performed the expression analysis of the class A, B, and C genes. A dramatic reduction of transcript levels of class B genes (*AP3* and *PISTILLATA*) was observed. Additionally, we also analyzed the methylation of the promoters of class B genes and found that the promoter of *AP3* was hypermethylated. Furthermore, combining mutations in *rdr2-2*, *drm1/2*, and *nrpd1b-11* with the *AP3*-silencing lines rescued the abnormal development of stamens and petals. The expression of *AP3* was reactivated and the methylation level of *AP3* promoter was also reduced in RdDM-defective *AP3*-silencing lines. Our results showed that the RdDM pathway contributed to the transcriptional silencing in the transgenic *AP3*-silencing lines. Moreover, the results revealed that fact that the exogenous fragment of a promoter could trigger the methylation of homologous endogenous sequences, which may be ubiquitous in transgenic plants.

## 1. Introduction

T-DNA insertion mutants of *Arabidopsis* are invaluable resource for studies of gene functions. [1]. However, the silencing of 35S promoter-driven transgenes might occur in T-DNA (*SALK, GABI*, and *FLAG*) insertion mutants [2,3]. Studies have shown that some T-DNA transgene silencing of the 35S promoter insertion is short-interfering RNAs (siRNA)-mediated [2]. In transgenic plants, the expression of a target gene or an endogenous gene is inhibited by the transfer of a foreign gene into a plant, but usually only affects the transferred gene and its endogenous gene and does not affect the expression of other genes. Plant transgenic silencing was first discovered in 1990 [4]. As the research has progressed, it is found that transgene silencing is universal and can even affect plant development.

Transgenic silencing triggered by epigenetic modifications in plants can be affected by small RNAs (sRNAs), both directly and in a mediated manner. sRNAs may mediate post-transcriptional gene silencing (PTGS), which is associated with the impairment of target RNA, and thus play an essential role in epigenetic gene regulation [5]. In plants, sRNAs are derived from cleavage of double-stranded RNA (dsRNA), which may trigger PTGS, and dsRNA can be made in the nucleus or cytoplasm. The dsRNA might originate from the transcription through IRs (inverted DNA repeats), an unlinked homologous transcribed locus, or an exogenous source, such as a single-stranded RNA (ssRNA) template catalyzed by RNA-dependent RNA polymerases (RdRP) [6]. Then, dsRNAs are processed into sense and antisense RNAs 21–25 nt long by RNase III-type protein termed Dicer, and the antisense small interfering RNA (siRNAs) are loaded into an argonaute family protein (ARGONAUTE 1 (AGO1)) to form RNA-induced silencing complex (RISC), which interacts with the homologous single-stranded mRNAs by pairing with the antisense siRNAs and then cleaves the mRNAs. The mRNAs cleaved by RISC may also be used as templates to form dsRNA, resulting in enhanced PTGS by increasing the level of siRNAs. Moreover, sRNAs produced in the cytoplasm can also enter the nucleus and trigger homologous DNA methylation [7]. AGO1 mainly acts in siRNA pathways for post-transcriptional gene silencing (PTGS), whereas AGO4 regulates transcriptional gene silencing (TGS). AGO4 regulates epigenetically silent states of repeated loci, transposons, and heterochromatin regions through its associated 24-nucleotide (nt) siRNAs [8]. RdDM is a key factor for the artificial induction of TGS [9]. Meanwhile, RdDM might be required for the initiation or maintenance of transcriptional silencing. Cytosine DNA methylation is a stable epigenetic mark and is important in many processes, including genomic imprinting, the silencing of transposons and genes, X chromosome inactivation, and paramutation in plants [10,11].

In *Arabidopsis*, DNA methylation occurs frequently in all three sequence kinds: The symmetric CG, CHG contexts, and the asymmetric CHH context (in which H = A, T, or C). The establishment of DNA methylation is through the RdDM pathway [12]. *Arabidopsis* has at least three classes of DNA methyltransferase genes that are possibly involved in controlling RdDM: The DNA methyltransferase (MET) class, the chromomethylase (CMT) class, and the domains rearranged methyltransferase (DRM) class, among which MET1 maintains CG methylation [13], CMT3 maintains CHG methylation [14], while DRM2 catalyzes the de novo cytosine methylation in all three sequence kinds [15,16]. In the RdDM pathway, both 24nt siRNAs and long noncoding RNA transcripts are essential for de novo DNA methylation [11]. RNA Pol IV and Pol V, which respectively participate in catalyzing of the production of 24 nt siRNAs and long noncoding RNA, have a distinct largest subunit (nuclear RNA polymerase D1 (NRPD1) and nuclear RNA polymerase E1 (NRPE1), respectively) while share some common subunits with Pol II [17,18]. siRNAs are produced from dsRNAs. Pol IV is recruited to transcribe transposons or some endogenous repeat loci, and the transcripts copied into dsRNAs by RNA-dependent RNA polymerase (RDR2) [19]. Dicer-like 3 (DCL3) cleaves the dsRNA into 24 nt siRNA duplexes and the siRNAs are subsequently methylated at their 3′ ends by the RNA methylase HUA-enhancer 1 (HEN1) for stabilization, and then one strand of the siRNAs is loaded into AGO4 to form an RISC [20]. On the other hand, Pol V is thought to transcribe the intergenic non-coding regions throughout the genome, the transcription is assisted by the DDR complex, including DRD1 (defective in RNA‑directed DNA methylation 1), DMS3 (defective in meristem silencing 3), RDM1 (required for DNA methylation 1) and DMS4 [21,22]. RISC interacts with the Pol V subunit NRPE1 through the base-pairing of 24 nt siRNAs with the nascent Pol V transcripts from the target loci [23,24], and then DRM2 is recruited to the region, which results in DNA methylation [23,25].

In *Arabidopsis*, class B genes are represented by *AP3* and *PI*, which control the development of petals and stamens. Class B mutants develop sepals instead of petals in the second whorl, developing carpels rather than stamens in the third whorl [26,27,28]. *AP3* contributes to the petal and stamen primordia, and the expression of *AP3* is maintained in these two parts during the subsequent floral development. Spatial and temporal restriction of *AP3* transcription is controlled via the interactions between proteins binding to different domains of the *AP3* promoter [29]. In *Arabidopsis*, transgene silencing often occurs when silencing constructs are introduced (self-compatible IR or antisense (AS) orientations) or some T-DNA mutant lines are used. In our study, the transgenic lines (without IR or AS constructs) transformed from wild-type *Arabidopsis* also displayed an occurrence of transcriptional silencing.

To study the mechanism of tribenuron-methyl (TM)-induced male sterility in *Arabidopsis* and rapeseed (*Brassica napus*), we expressed TM resistance genes *CYP81A6* [30] and *csr* (*ALS* point mutant) [31] under several promoters (unpublished). The majority of transgenic lines showed wild-type phenotype without TM treatments. Nevertheless, some of transgenic lines containing genes driven by the *AP3* promoter showed some morphological defects, including lack of stamens and petals in *Arabidopsis* and wizened and smaller petals in rapeseed, which motivated the present study. To determine the mechanism underlying the defective phenotypes, we conducted a series of detailed biochemical and genetic analyses in *Arabidopsis*. The results show that the expression of *AP3* is correlated with the hypermethylation of the *AP3* promoter mediated by the RdDM pathway in the transgenic plants of *Arabidopsis*.

## 2. Results

### 2.1. Defective Phenotypes Observed in the Transgenic Lines

**Figure 1:**
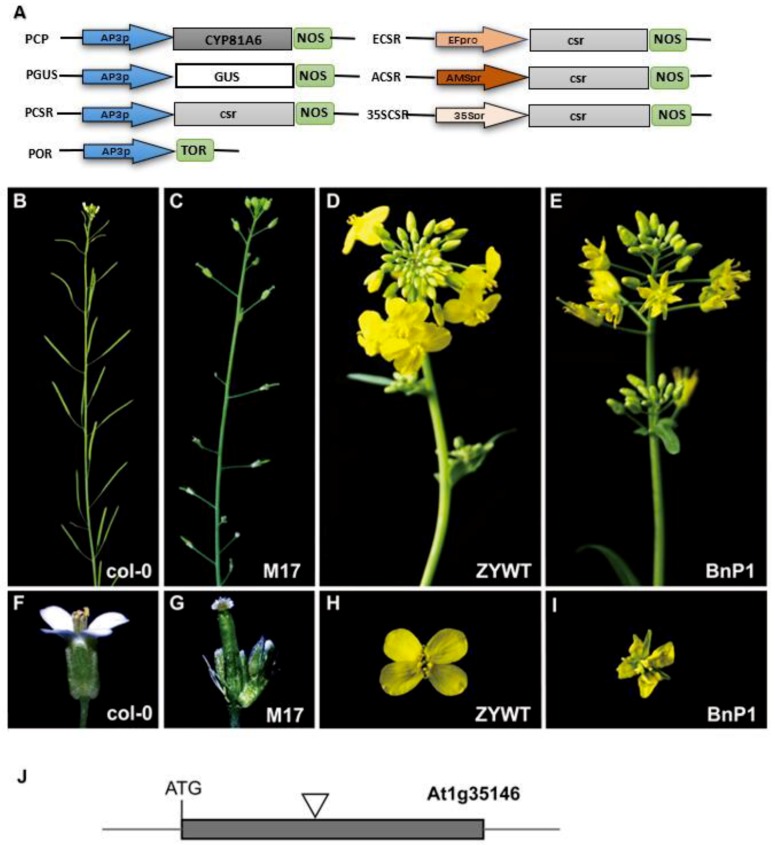
(**A**) Schematic illustration of constructs used to transform *Arabidopsis* and rapeseed. Arrows indicate the promoters. The fragments were cloned to the expression sites of *pCAMBIA2300*; (**B)** feature of a wild silique of *Arabidopsis*; (**C**) feature of a male sterile transgenic line without petals and stamens of *Arabidopsis*; (**D**) rapeseed with normal inflorescence; (**E**) transgenic inflorescence of rapeseed (BnP1) with defective petals; (**F**) a wild flower with normal petals of *Arabidopsis*; (**G**) a male sterile transgenic *Arabidopsis* flower without petals and anthers on top of a filament; (**H**) a wild flower of rapeseed; (**I**) a transgenic flower of rapeseed (BnP1) with wizened petals; (**J**) DNA insertion occurred in the exon of At1g35146 based on the results from TAIL-PCR analysis. Grey box: Exon; grey lines: Intergenic sequences; black triangles: Location of transgenes.

To investigate the contribution of tissue-specific expression of TM resistance genes for male sterility, we generated transgenic *Arabidopsis* lines carrying an insertion fragment (PCP or PCSR), which contained *CYP81A6* or *csr* (dominant mutation of CSR) genes under the control of the *Arabidopsis AP3* promoter (Figure 1A, Supplementary Figure S5). *Arabidopsis thaliana* transgenic materials M17 and M43 (Table S2) and rapeseed transgenic materials BnP1, BnP2, BnP3, BnP4, and BnP5 were used as experimental materials in this study. In *Arabidopsis* with M17 as a phenotype, as shown in Figure 1C,G, compared with wild type (Figure 1B,F), the main inflorescences were not elongated, and the second round of petals and the third round of stamen development were defective. For example, BnP1 in *Brassica napus L*. (Figure 1E,I) showed no significant difference in the overall appearance of the inflorescence compared to the wild type (Figure 1D,H), but the flowers were small and the petals were small and shrunk. We designated the abnormal phenotypes, which were similar to that of *ap3-3* mutant, as *ap3*-like phenotypes. T2 plants (*ap3*-like phenotypes) were genotyped by genomic PCR. The results demonstrated that half of the plants harbored the insertion fragments and showed *ap3*-like phenotypes defects. By cross pollination with pollens from wild-type *Arabidopsis* flowers, Some of the transgenic lines of rapeseed, which also contained PCP or PCSR constructs, showed similar defects, such as shriveled and smaller petals, less fertile stamens, which produce lesser pollen, and smaller siliques compared with ZYWT (wild type) (Figure 1E,I). We used the TAIL-PCR technique to perform flanking sequence analysis on the transgenic plant M17. According to the results of the flanking sequence analysis, it was found that the exogenous fragment was inserted into the exon of At1g35146, and the subsequent PCR identification of the M17 progeny was based on this.

**Figure 2:**
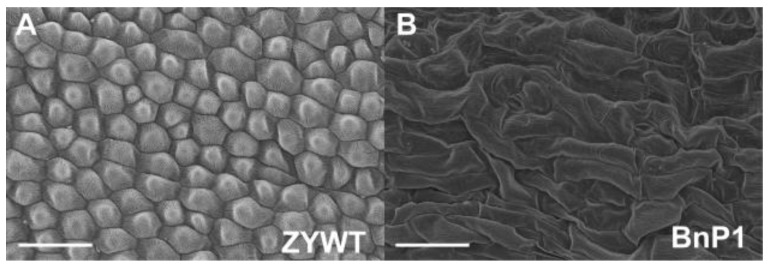
Scanning electron micrograph. (**A**) A wild flower with normal petals of *B. napus*; (**B**) a transgenic line (BnP1) with irregularly shaped cells in petals. Bars = 50 µm.

In BnP1 (containing PCSR construct) flowers, scanning electron micrograph showed that the petals had irregularly shaped but elongated epidermal cells and stomas (Figure 2B), while the cells in wild plant flowers were well-organized and closely arranged (Figure 2A).

### 2.2. Exogenous AP3 Promoter was Responsible for the ap3-Like Phenotypes

To investigate the association of this chimeric gene with the *ap3*-like phenotypes, the PGUS construct that contained the β-glucuronidase (GUS) and the construct (POR) without genes driven by the *AP3* promoter were prepared and transformed into *Arabidopsis*, respectively (Figure 1A). Some of both PGUS and POR transgenic lines showed *ap3*-like phenotypes, indicating that these chimeric genes (the TM resistance genes, the *GUS* gene, or even no gene driven) were not the major controlling elements.

It was unclear whether the vector or selectable marker was responsible for the *ap3*-like phenotypes. Hence, we cloned the PCSR fragment into *pCAMBIA1300* and *PBI121* vectors. Some of both the two transgenic lines showed the *ap3*-like phenotypes. We also transformed the *pCAMBIA2300* vector into *Arabidopsis* plants, but the transgenic lines did not show any morphological defects. In order to exclude the possibility that the selectable marker was responsible for the phenotypes, the PGUS fragment was transformed into *Arabidopsis* plants using the double right-border (DRB) T-DNA binary vector that was provided by Prof Yongjun Lin (National Key Laboratory of Crop Genetic Improvement, Huazhong Agricultural University, Wuhan, China), to generate marker-free transgenic lines. Seventeen of 28 transgenic lines displayed *ap3*-like phenotypes. The sterile transgenic lines were crossed with the wild type to generate posterity lines, which may have contained the PGUS or the selectable marker gene fragment, respectively. Only lines that carried the PGUS fragment showed *ap3*-like phenotypes. These results suggested that the vector or selectable marker was not responsible for the *ap3*-like phenotypes.

From these transformation results, we found that all the transgenic lines with *ap3*-like phenotypes may contain the *AP3* promoter (Supplementary Figure S4). To determine whether the *AP3* promoter was the major controlling element for the morphological defects, a series of constructs were generated and transformed into *Arabidopsis* plants (Figure 1A). The constructs contained *csr* driven by the 35S (cauliflower mosaic virus), *EF*, or *AMS* promoter, and all the transgenic lines did not show any *ap3*-like phenotypes, suggesting that it was the *AP3* promoter rather than the 35S, *EF*, or *AMS* promoter that was the major controlling element for *ap3*-like phenotypes.

### 2.3. ap3-Like Phenotypes were Correlated with the DNA Hypermethylation of AP3 Promoter

**Figure 3:**
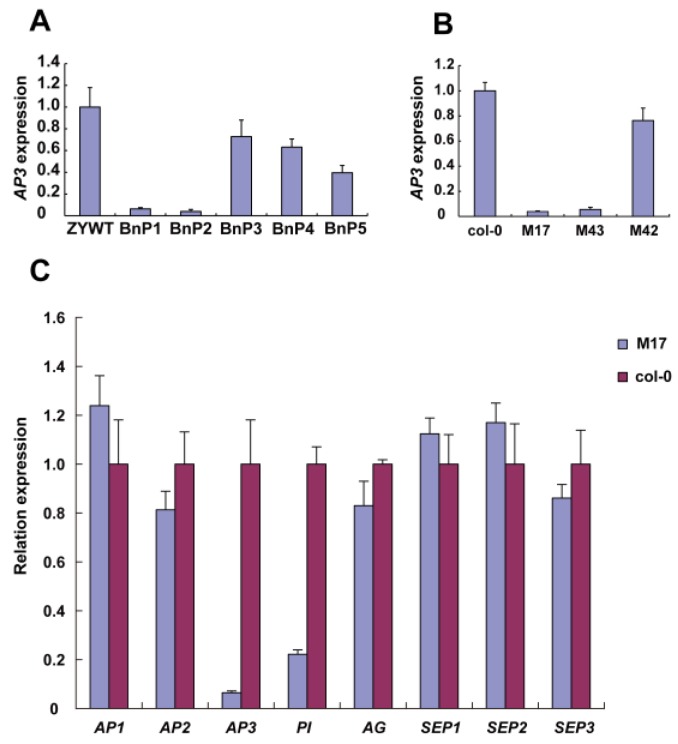
RT-PCR analysis of selected target genes. (**A**) *AP3* expression of transgenic rapeseed BnP1 and BnP2 with severe morphological defects and BnP3, BnP4, and BnP5 with slight morphological defects, ZYWT (wild-type); (**B**) *AP3* expression of transgenic *Arabidopsis* M17, M43 with morphological defects, and M42 with no defects, Col-0 (wild plant)(http://katahdin.mssm.edu/kismeth/revpage.pl) [32,33,34]; (**C**) the levels of class A, B, and C transcripts. All the rapeseed (P1–P5) and *Arabidopsis* (M17, M42, M43) transgenic lines contain the PCSR construct. The expression levels were controlled using the signal from the *ACTIN* gene. The average (±SD) values are shown from three biological duplicates of quantitative reverse transcription PCR.

In comparison with Col-0 and M42, M17 and M43 showed a sharp decline of *AP3* expression. Additionally, we compared the *AP3* expression in rapeseed (petals) and a similar result was obtained. The phenotypic defects of BnP1 and BnP2 are more serious, while the phenotypes of BnP3, BnP4, and BnP5 are weaker, and the expression of *AP3* gradually decreases with the severity of phenotypic defects (Figure 3A). To examine the cause of the *ap3*-like phenotypes in the *Arabidopsis*, as the transgenic results showed that it was the *AP3* promoter that was responsible for the *ap3*-like phenotypes, we wanted to confirm that whether the *AP3* expression (inflorescences) was changed in the *ap3*-like phenotypes transgenic lines of *Arabidopsis*; therefore, we carried out expression analysis of *AP3* in wild-type *Arabidopsis* plants (Col-0), in M17 and M43 (transgenic lines with obvious morphological defects) and in M42 (transgenic lines without any defects) (Figure 3B).

Furthermore, real-time RT-PCR was carried out to determine the expression of class A, B, and C genes in *Arabidopsis* (inflorescences). The results show that for class A and C genes, no expression difference was observed between M17 and Col-0 (Figure 3C), while *AP3* and *PI* showed a sharp decrease of expression in M17. These results suggest that the decrease of *AP3* and *PI* expression might be responsible for the observed phenotypes. To determine whether the genes around the *AP3* region were influenced in the transgenic lines with *ap3*-like phenotypes, we compared the expression levels of these genes between Col-0 and M17 flowers (Supplementary Figure S1). The data suggest that there is no difference in gene expression between them. Together, these results indicate that it is class B homeotic genes rather than class A or C genes that are necessary for the development of sepals and petals, and the genes around *AP3* are not influenced in the transformation.

**Figure 4:**
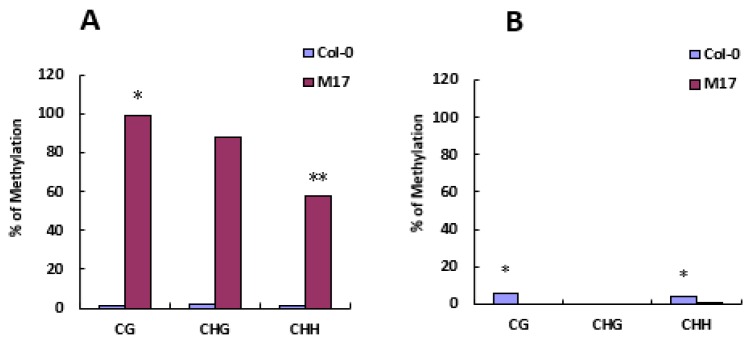
DNA methylation analysis of Col-0 and M17 by bisulfite sequencing. (**A**) DNA methylation analysis of *AP3* promoter (**B**) DNA methylation analysis of *PI* promoter. The percentage of methylation on CG, CHG, and CHH sites are shown and T test analysis of methylation differences in the group (* *p* < 0.05, ** *p* < 0.01).

Based on the above results, we speculated that the *ap3*-like phenotypes were triggered by epigenetic modifications. In order to clarify the cause of the morphological defects, we performed the DNA methylation analysis of *AP3* promoter in Col-0 and M17 by bisulfite sequencing in *Arabidopsis* (inflorescences). The results showed that the DNA methylation of *AP3* promoter was detected at low levels in Col-0, while a high level of DNA methylation at all sequence contexts (CG, CHG, and CHH) was found in M17 (Figure 4A). The DNA methylation level of *AP3* promoter was less than 50% when only one of the *AP3* promoters (exogenous and endogenous) was methylated, while the results showed that the methylation all of CG, CHG, and CHH sites covered 50%, so both exogenous and endogenous *AP3* promoters were hypermethylated, which is consistent with *ap3*-like phenotypes and the *AP3* expression of M17. Additionally, we also performed DNA methylation analysis on the *PI* promoter. The results suggest that DNA methylation of *PI* promoter was not observed at all three cytosine contexts in M17 and Col-0 (Figure 4B), which is opposite to the level of *PI* expression. The probable reason for the lack of *PI* transcripts in the flowers with *ap3*-like phenotypes was that the *AP3* expression is absent. We also found that the initial pattern of *PI* expression was normal in *ap3-3* flowers; thus, high levels of *PI* RNA are not maintained after stage 4 or 5 in *ap3-3* flowers. The results show that both *AP3* and *PI* are involved in the regulation of *PI* expression, whereas neither is required for the initiation of *PI* expression [35]. Taken together, these results show that the DNA methylation of *AP3* promoter is responsible for the defective flower development in M17.

**Figure 5:**
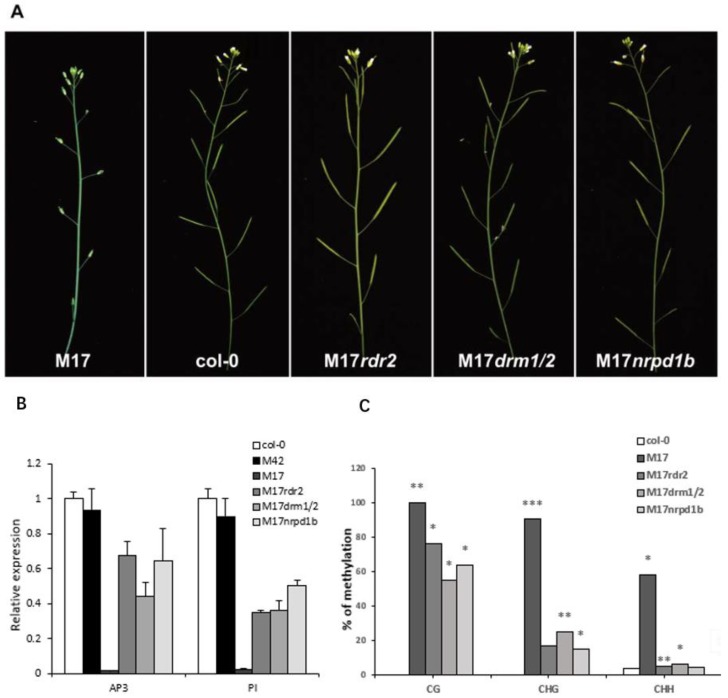
Complementation test for M17 transgenic lines. (**A**) All the morphological defects including sepaloid petals and absence of anthers in M17 were rescued in complementation lines; (**B**) RT-PCR analysis of the expression of *AP3* and *PI* in M17 and complementation lines; (**C**) cytosine DNA methylation analysis of transgenic, which is endogenous *AP3* promoters in M17 and complementation lines by bisulfite sequencing and T test analysis of methylation differences in the group (* *p* < 0.05, ** *p* < 0.01, *** *p* < 0.001). The expression levels were controlled using the signal from the *ACTIN* gene. The average (±SD) values are shown from three biological duplicates of quantitative reverse transcription PCR.

To further confirm that the DNA methylation of *AP3* promoter is responsible for the *ap3*-like phenotypes in M17, a complementation test was conducted as follows: M17 was crossed with null mutants *sgs3-11* and *rdr6-11* as SGS3 and RDR6 are required for PTGS [36]; M17 was also crossed with *rdr2-2*, *drm1/2*, and *nrpd1b-11*, which release the silencing of a reporter gene through the RdDM pathway. Among the examined F1 plants, lines with the PCSR construct showed the same phenotypes as M17. Then, double heterozygous plants (identified by genomic PCR) were chosen to be pollinated with pollens from the null mutants. In the BC1 generation, the morphological defects were fully complemented only in the lines with *rdr2-2*, *drm1/2*, and *nrpd1b-11* as the pollen donors (Figure 5A), whereas those lines containing *rdr6-11* and *sgs3-11* homozygote were not recovered due to fact that SGS3 and RDR6 were not present in the pathway of RdDM. We also analyzed the *AP3* expression, *PI* expression, and DNA methylation levels of *AP3* promoter by bisulfite sequencing in the complementation lines. In comparison with M17, the high methylation of the *AP3* promoter was substantially decreased at all the three cytosine contexts (CG, CHG, and CHH), and the reduced expression of *AP3* and *PI* were rescued in the complementation lines (Figure 5B,C). These results suggest that the morphological defects were controlled by RdDM. It was possible that the transgenic construct of *AP3* promoter may have generated siRNA and caused the DNA methylation of the *AP3* promoters, resulting in the decrease of *AP3* expression and obvious defects in floral morphology.

## 3. Discussion

### 3.1. Exogenous AP3 Promoter may Trigger the Hypermethylation of AP3 Promoter Mediated by RdDM in Transgenic Arabidopsis Plants

Epigenetic silencing is important for gene regulation during plant development and for the inactivation of viruses, transposons, or genes. DNA methylation is an important epigenetic marker conserved in plants, mammals, and some fungi for transcriptional gene silencing (TGS) in diverse organisms [37,38]. De novo cytosine methylation can be catalyzed in a pathway known as RdDM. The RdDM pathway is of central importance to the initiation and maintenance of TGS in plants [10]. In this study, our analysis of the DNA methylation in the *AP3* promoter of the transgenic *Arabidopsis* plants show that the transgenic homologous *AP3* promoter affected the overall methylation of exogenous and endogenous *AP3* promoters, as indicated by the strong correlation between the decrease of *AP3* transcripts in transgenic lines with *ap3*-like phenotypes and a high level of overall methylation of *AP3* promoter (Figure 3 and Figure 4), and the transformation results in *Arabidopsis* indicated that in transgenic lines it was the *AP3* promoter triggering the *ap3*-like phenotypes. These results suggest that the exogenous *AP3* promoter plays an important role in regulating the TGS of *AP3*. However, gene-silencing phenomena might occur at the post-transcriptional level, which involved mRNA specific degradation in the cytoplasm or the methylation of coding sequences [39,40]. Several reports have shown that PTGS can not only affect transgenes that are homologous to endogenous genes, but also transgenes that are not homologous to endogenous genes, suggesting that the PTGS is not a simple regulatory mechanism affecting endogenous gene expression [41,42,43]. We also speculated that the decrease of *AP3* expression is related to PTGS. SGS3 and RDR6 are required for the biosynthesis of trans-acting siRNAs, and they initiate PTGS or trigger DNA methylation [36,44]. However, BC1 transgenic lines that contained *sgs3-11* or *rdr6-11* homozygote showed no morphological alterations. On the other hand, the BC1 transgenic lines with *rdr2-2*, *drm1/2*, and *nrpd1b-11* as the pollen donors showed no defects in floral morphology as well as a reduced the DNA methylation level (Figure 5). These findings suggest that RdDM is required for the silencing of *AP3*. Furthermore, the phenotype of the complementation lines containing *rdr6-11* and *sgs3-11* homozygote was not recovered, suggesting that the silencing of *AP3* is not related to PTGS.

PTGS and TGS were considered to be the major pathways of epigenetic regulation, and both of them might be associated with methylation. Recent studies have already indicated that transgenes silencing might accompany methylation, transgenic lines of *Nicotiana attenuata* displayed a variable occurrence of transgene silencing, and bisulfite sequencing analysis indicated a hypermethylation within the *35S* and *NOS* promoters of these lines [45]. The RNA interference (RNAi) induces gene silencing accompanied with methylation by an IR directed at the promoter or coding sequence [46]. The hypermethylation of *AP3* promoter found in M17 containing an extra copy of *AP3* promoter was consistent with those who found that unwanted transgenes silencing was commonly associated with hypermethylation within the promoter regions of transgene (Figure 4A).

### 3.2. The ap3-like Phenotypes was Inherited

We combined phenotypes of transgenic plant by crossing transgenic line (M17) and wild-type, the offspring of which harbored the transgenes that showed *ap3*-like phenotypes, suggesting that the transgenic silencing might be inherited, similar as reported for *N. attenuate*, which showed loss of hygromycin resistance and a drastic down-regulation of antimicrobial peptide gene expression in the T3 generation of transgenic *N. attenuate* [45]. However, the gene inactivation that occurred in plants with multicopy integration of the foreign DNA and transgene silencing would be reactivated by crossing with wild-type plants [47]. In our case, the *ap3*-like phenotypes did not change in the offspring of several regenerants, probably because the methylation levels had already accumulated in T1 plants and the foreign *AP3* promoter existing in the offspring might trigger the de novo cytosine methylation.

### 3.3. The Characteristic of AP3 Promoter Methylation in Plants with ap3-Like Phenotypes

It was previously reported that TGS is induced by RdDM following methylation that spreads from the originally targeted region to the adjacent regions [48]. In contrast, in M17, the genes around the *AP3* region did not show any change in expression level (Supplementary Figure S1). These results suggest that the extraneous fragment only influences the TGS of homologous regions but does not affect that of nonhomologous regions. TGS with methylated promoters frequently have tissue-specific expression patterns [49]. Nevertheless, in our study, methylated *AP3* promoter did not show tissue-specific patterns, as indicated by the DNA methylation analysis using leaves, stems, and inflorescences, all of which showed hypermethylation in *AP3* promoter sequences (Supplementary Figure S2). Direction of RdDM to a promoter sequences can lead to the occurrence of TGS. These data suggest that the high DNA methylation of *AP3* promoter has an obvious effect on the regulation of *AP3* gene transcription and the *ap3*-like phenotypes (Figure 2B and Figure 3A).

Our discovery of the way that exogenous fragment triggers DNA methylation of homologous sequences may be extended to all other transgenic events. The phenomenon occurred not only in *Arabidiopsis*, but also in rapeseed in our transgenic experiments (Figure 1B–I and Figure 2A,B), indicating that it may occur in more plants. Exogenous fragment in plants may trigger the silencing of endogenous sequences.

### 3.4. An Objective View on Transgenes Silencing

As our results indicate, the extraneous *AP3* promoter has an important role in gene silencing, which is controlled by RdDM. In many transgenic studies, some unexpected phenotypes derived from TGS might be obtained. A previous study has shown that transcriptional silencing was mediated by the 35S promoter homologous between transgenes and the T-DNAs (such as the SALK, FLAG, and GABI) used for insertional mutagenesis [3]. Transgene silencing phenomena may occur when there are multiple copies of a particular sequence in a genome and the silencing results from interactions between homologous sequences [50,51]. In our studies, transcriptional silencing was accompanied by hypermethylation in the *AP3* promoter in transgenic *Arabidiopsis* plants, similar to reports that silencing often occurs when the genome existed in the transgene, which was homologous to endogenous sequences [50]. So, care must be taken to control for unwanted silencing when studying expression of transgenes, as it might trigger transgene silencing when the transgenic construct used and the genome share regions of homology. More molecular biological analyses should be carried out to determine whether exogenous fragments induce TGS of the homologous regions in plants. When the transgenic lines display unwanted or unexpected phenotypes, it does not necessarily mean that the gene has no function as the genes cannot be transcribed normally. In transgenic studies, it is imperative to conduct more experiments to determine whether the construct is stably transformed into the plants; real-time RT-PCR analysis should be conducted to analyze the gene expression and Southern blotting should be taken to detect the insertion number, and more complementation tests should be conducted to confirm whether the gene silencing is responsible for the observed phenotypes.

## 4. Materials and Methods

### 4.1. Plant Materials

The mutant alleles of *rdr2-2*, *drm1/2*, *nrpd1b-11*, *rdr6-11*, and *sgs3-11* [44] used in this study were in a Columbia (Col-0) genetic background. M42, M43, and M17 were the transgenic *Arabidopsis* lines with the PCSR constructs containing the 1.2 Kb *AP3* promoter. Plants were grown in a growth chamber or controlled temperature at 23 °C with 16 h of light and 8 h of darkness. The rapeseed (cv. Zheyou 50) was grown in soil under natural conditions. BnP1, BnP2, BnP3, BnP4, and BnP5 were transgenic lines (contain PCSR constructs) of rapeseed.

### 4.2. Vector Construction and Expression of csr or CYP81A6 in Arabidiopsis and Rapeseed

The *AP3, EF*, and *AMS* promoter fragments were amplified from *Arabidiopsis*. The cauliflower mosaic virus (CaMV) 35S promoter was amplified from *pCAMBIA2300*. The products were cloned into the *pCAMBIA2300*, *pCAMBIA1300*, or *PBI121* vector. All binary vector expression constructs were transferred to *Agrobacterium* tumefaciens strain *GV3101* by electroporation and were transformed into Col-0 and Zheyou 50 plants using the methods previously described [52].

### 4.3. Scanning Electron Micrograph

SEM was used to study the surface of petals of the wild-type and transgenic rapeseed plants. Fresh petals from 4–7 mm buds were collected and fixed in 2% glutaraldehyde overnight. The ensuing procedures were performed as previously reported [53].

### 4.4. RNA Isolation and Real-Time RT-PCR

Total RNA was isolated from inflorescences (*Arabidopsis*) and petals (rapeseed) using Trizol (Bioteke, Beijing, China) according to the manufacturer’s instructions. DNase treatment was performed on total RNA to remove the genomic DNA contamination, and then the RNA was used for first strand cDNA synthesis using a SuperScript II kit (Invitrogen, Carlsbad, CA, USA) following the manufacturer’s protocol with an oligo(dT)_18_ primer. The derived cDNA was used as template for real-time RT-PCR analysis. Quantitative PCR was performed with SYBR Green Realtime PCR Master Mix (TOYOBO, Osaka, Japan). *ACTIN* mRNA was detected in parallel and controlled data normalization. The primers used for quantitative PCR are listed in Supplementary Table S1.

### 4.5. DNA Methylation Analysis

Fresh tissues (inflorescences, stems, and leaves) were collected for genomic DNA isolation. DNA methylation analysis was performed by the bisulfite sequencing method [54]. The DNA was isolated using the DNeasy Plant Mini Kit (Qiagen, Hilden, Germany). Bisulfite sequencing was performed, and 600–800 ng of DNA was sodium-bisulfite converted and purified using the EpiTect Bisulfite Kit (Qiagen, Hilden, Germany) according to the manufacturer’s instructions. Treated DNAs were used for PCR amplification of different loci using rTaq DNA polymerase (Takara, Tokyo, Japan), PCR conditions were as follows: 94 °C, 5 min; 38 cycles of (94 °C, 45 s; 48 °C, 30 s; 72 °C, 30 s); 72 °C, 10 min. The PCR products generated using two primer pairs from the promoters of *AP3* and *PI* were of identical size (the −486 to −228 promoter fragment of *AP3* and −794 to −465 promoter fragment of *PI*) in M17 and Col-0. PCR products were cloned into the pMD18-T vector (Takara, Tokyo, Japan), and individual clones were sequenced. For the DNA methylation analysis, 6–8 clones of each sample were sequenced. Data analysis and primers design were performed using the online tool. The degree of conversion was determined by sequencing a region of the *PHAVOLUTA* locus [55]. The bisulfite conversion rate for the sample was over 99%. Primers used for bisulfite sequencing are described in Supplementary Table S1.

### 4.6. Transgenic and SALK Lines Analyses

Homozygous SALK lines were identified by PCR genotyping to determine whether T-DNA insertion exists. Total genomic DNA samples were extracted using the fresh leaves of plants. TAIL-PCR technique was used to determine the insertion position of the PCSR construct in M17 (At1g35146) (Figure 1I). We designed the primers for identifying the transgenic lines: One primer specific to the region around the inserted fragment, and one corresponding to a constitutively expressed kanamycin gene. The plants of BC1 generation were genotyped by genomic PCR, and plants harboring the PCSR construct and the T-DNA insertion from null mutants were subjected to morphological observations, real-time RT-PCR, and DNA methylation analysis. Progenies of this backcross were allowed to self-pollinate. The primers used for transgenic lines analysis are listed in Supplementary Table S1.

## 5. Conclusions

Through all of the above experiments, we revealed the fact that exogenous fragments of the promoter can trigger methylation of homologous endogenous sequences, which may be ubiquitous in transgenic plants.

## Supplementary Materials

Supplementary materials can be found at https://www.mdpi.com/1422-0067/20/18/4478/s1.
