# Supplementary material for: Exogenous Promoter Triggers APETALA3 Silencing through RNA-Directed DNA Methylation Pathway in Arabidopsis
Source: Int J Mol Sci. 2019 Sep 11;20(18):4478. doi: 10.3390/ijms20184478 (PMC6770043; doi:10.3390/ijms20184478)
Supplement: Supplementary file 1 [file ijms-20-04478-s001.zip › ijms-573278 Suppl for final/ijms-573278-supplementary Figure for final.pdf]

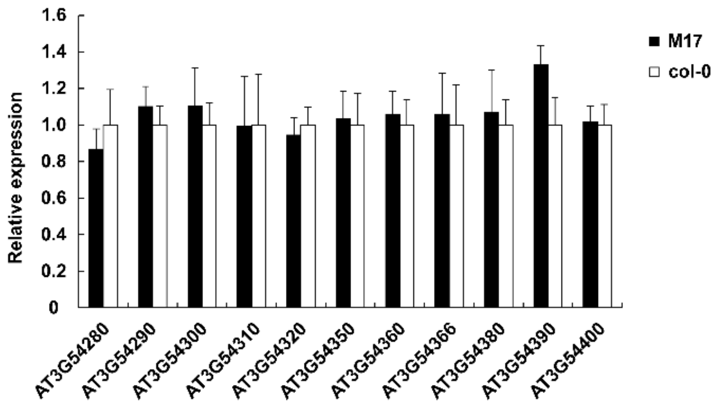

Supplementary Figure S1. The expression levels of these genes between col-0 and M17 flowers.

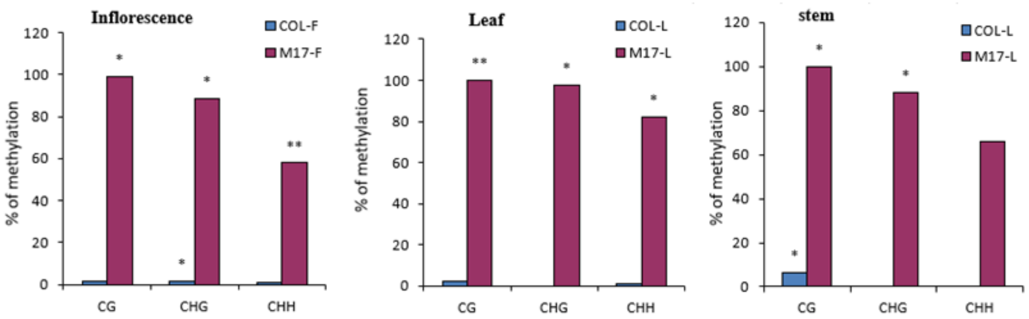

Supplementary Figure S2. DNA methylation analysis using leaves, stems and inflorescences. T test analysis of methylation differences in the group.

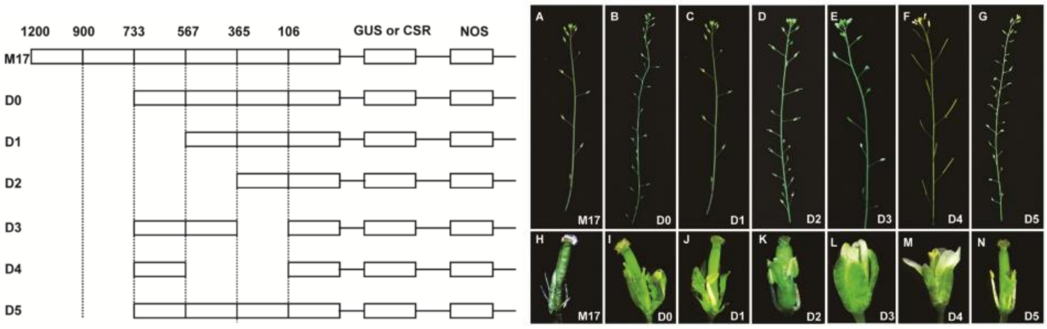

Supplementary Figure S3. *AP3* promoter truncation experimental vector schematic and transgenic phenotype.

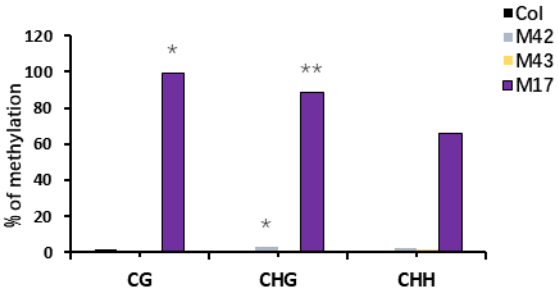

Supplementary Figure S4. *AP3* promoter DNA methylation analysis M17, M42, M43, Col-0. T test analysis of methylation differences in the group

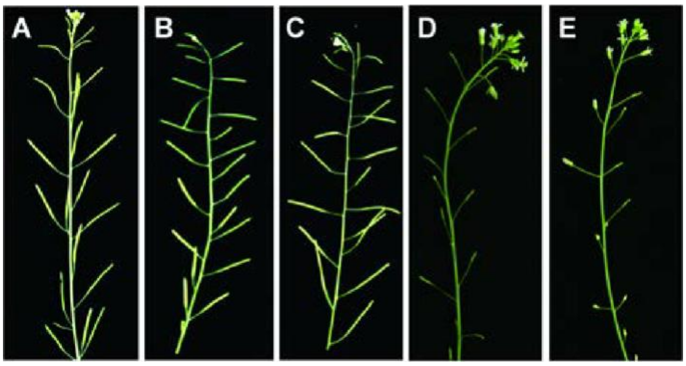

Supplementary Figure S5. Other transgenic phenotypes of the *ap3*-like phenotype. (**A**): 35SCSR; (**B**): ECSR; (**C**): ACSR; (**D**): PCP; (**E**): PCSR.
