# Supplementary material for: Exogenous Promoter Triggers APETALA3 Silencing through RNA-Directed DNA Methylation Pathway in Arabidopsis
Source: Int J Mol Sci. 2019 Sep 11;20(18):4478. doi: 10.3390/ijms20184478 (PMC6770043; doi:10.3390/ijms20184478)
Supplement: Supplementary file 1 [file ijms-20-04478-s001.zip › ijms-573278 Suppl for final/ijms-573278-Supplementary table 1 The primers used for quantitative PCR.pdf]

Supplementary table 1 The primers used for quantitative PCR

Name	Sequence
AP3-L1-qRT	CGAGACCACCAAGAAAAAGAACAA
AP3-R1-qRT	GGTGGAAACGAAGAGCGTAAGC
SEP3-L-qRT	ATGCTTCGGACACTGGAGAGGTA
SEP3-R-qRT	GCTTGAGATACTCCTGCTGGCTAC
SEP2-L-qRT	TGGACATCCTCAGGCTCATTCTC
SEP2-R-qRT	ATGTAGCCGTTTCCTTGTTGGG
SEP1-L-qRT	GATGGGAAGGTGGTGAACAGAATG
SEP1-R-qRT	GCTTGGGTTGTCGCAGTTATTTG
AG-L-qRT	CCACCTCAAACGCAATCTCAACC
AG-R-qRT	CACTAACTGGAGAGCGGTTTGGTC
PI-L-qRT	ACTTGAAAAATCTGATGGCTGTCG
PI-R-qRT	GCTGGAAAGTGAGTTGCCGTTG
AP2-L-qRT	ATCAAATGTAACGGCAAAGACGC
AP2-R-qRT	TGGTTCATCCTGAGCCGCATA
AP1-L-qRT	CAGCACCAAATCCAGCATCCTTAC
AP1-R-qRT	AGCAGCCAAGGTTGCAGTTGTAAA
SPL-L-qRT	ACTCGGGAGCAACAGGATCTATTG
SPL-R-qRT	CACAGCGATTATTGGAAGAAGCG
AT3G54280-L-qPCR	AAAGGAATGAAGGCAATTCTAGGAAAC
AT3G54280-R-qPCR	CCACTACAAAAACTACTTCGGCGG
AT3G54290-L-qPCR	CGACGAAGCGACTCCATTGATAG
AT3G54290-R-qPCR	TCCTCCGACCATCTCAACATCCT
AT3G54300-L-qPCR	TTGACAAAACCGAGAACCTACAGT
AT3G54300-R-qPCR	GCAACAACCCAAACGATGAGAAT
AT3G54310-L-qPCR	ATTGTTTGTGGTGTTTGGCATACTG
AT3G54310-R-qPCR	AACACATTTCCTCTCCACATCTTCC
AT3G54320-L-qPCR	GATACAGGGTTTTCTCCGTTTTTG
AT3G54320-R-qPCR	ATCCAGATTTTCCAAGTTCAAGCA
AT3G54350-L-qPCR	ATACGGCTGGGTGATGACGGT
AT3G54350-R-qPCR	AAAGGCATTCCCCGTATCTCAAC
AT3G54360-L-qPCR	TTGGTGACTGTAGCCGAGCGAT
AT3G54360-R-qPCR	ATCTCCAAATCATTCAACGGCAG
AT3G54366-L-qPCR	TTCAACAATACGGATCTAGGGCG
AT3G54366-R-qPCR	AAAACCTTGGGATACTTTGGATGG
AT3G54380-L-qPCR	AGAACAGCTTCTGAGGCGACATAC
AT3G54380-R-qPCR	AGATAACCGCAAGAGAGGATAGGG
AT3G54390-L-qPCR	GGTGGTTAGCGGAGGTGGTGAT
AT3G54390-L-qPCR	TATCTCCGTCCGTTTCAAATCCA
AT3G54400-L-qPCR	ACTTGGTTGGGATTCGTGTCGG
AT3G54400-L-qPCR	AGCCTTGTGTAGACCGTCCCCG
**Primers used for bisulfite sequencing.**	
Me-L462	TTTTGTTTAYTTTAGGGTAAGTGTA
Me-R695	ATTACAARACACTRTTTAAATCAAC
PI-L558	TTGAAGAAAAYATAGTAYGGAAGAG
PI-R790	TCRCTTATTACACATARCCAACTCT

The primers used for transgenic line analysis

KLB1	GCTCTGATGCCGCCGTGTTCC
KLB2	CGGCGGCTGCATACGCTTGAT
KLB3	GCTGACCGCTTCCTCGTGCTTT
M17-L	GCTTACAACCTCTTCTGCCTCTA
M17-R	ATTGCCATTGCCCACTCTTT

Bisulfite sequencing of AP3 promoter
Bisulfite sequencing of AP3 promoter
Bisulfite sequencing of PI promoter
Bisulfite sequencing of PI promoter
