# Supplementary material for: Exogenous Promoter Triggers APETALA3 Silencing through RNA-Directed DNA Methylation Pathway in Arabidopsis
Source: Int J Mol Sci. 2019 Sep 11;20(18):4478. doi: 10.3390/ijms20184478 (PMC6770043; doi:10.3390/ijms20184478)
Supplement: Supplementary file 1 [file ijms-20-04478-s001.zip › ijms-573278 Suppl for final/ijms-573278-Supplementary table 2 Segregation analysis of fertility in the BC1F1 progenies.pdf]

Supplementary table 2 Segregation analysis of fertility in the BC1F1 progenies

plants	sterile	fertile	χ^2
M17	31	35	0.24(P>0.05)
M43	53	57	0.15(P>0.05)
